# Dieting status influences associations between dietary patterns and body composition in adolescents: a cross-sectional study

**DOI:** 10.1186/1475-2891-12-51

**Published:** 2013-04-24

**Authors:** Anna S Howe, Katherine E Black, Jyh Eiin Wong, Winsome R Parnell, Paula ML Skidmore

**Affiliations:** 1 Department of Human Nutrition, University of Otago, PO Box 56, Dunedin 9054, New Zealand; 2 School of Healthcare Sciences, Faculty of Health Sciences, Universiti Kebangsaan Malaysia, Kuala Lumpur 50300, Malaysia

**Keywords:** Dietary patterns, Body composition, Dieting status, Adolescents, New Zealand

## Abstract

**Background:**

Associations between food choice and body composition in previous studies of adolescents have been inconsistent. This may be due to the body composition measures used, or these associations may be affected by the dieting status of adolescents. The objective of this study was to investigate the association between dietary patterns and body composition in adolescents, and determine if these associations are moderated by dieting status.

**Methods:**

Information on food consumption and current dieting status was collected, using a web-based survey, in 681 adolescents (mean age 15.8 (SD 0.9) years) from schools in Otago, New Zealand. Non-dieters were defined as those reporting not being on a diet as they were “happy with their weight”. Principal components analysis (PCA) was used to determine dietary patterns. Body mass index (BMI), waist circumference (WC), waist-to-height ratio (WHtR), fat mass index (FMI), and fat-free mass index (FFMI) were examined as outcomes. Generalized estimating equations were used to examine associations between dietary patterns and body composition.

**Results:**

PCA produced three dietary patterns: ‘Treat Foods’, ‘Fruits and Vegetables’, and ‘Basic Foods’. A standard deviation increase in ‘Basic Foods’ was associated with a 3.58% decrease in FMI (95%CI −6.14, -0.94) in the total sample. When separate sex analysis was undertaken significant negative associations were found in boys only, between the ‘Basic Food’ score and WC, WHtR, FMI, and FFMI, while the ‘Fruits and Vegetables’ pattern was negatively associated with FMI. Associations between ‘Treat Foods’ and BMI, WC, and WHtR in non-dieters were positive, while these associations were negative for all other participants.

**Conclusions:**

Significant associations were found between dietary patterns and indices of both central and total adiposity, but not BMI. Therefore using only BMI measures may not be useful in this age group. Since our results were significant for boys and not girls, nutrition messages designed to prevent obesity may be particularly important for adolescent boys. As an interaction between dieting status and ‘Treat Foods’ existed, future studies should also explore the role of dieting when investigating food choice and body composition.

## Background

High rates of child and adolescent obesity are of concern worldwide and New Zealand is no exception, with at least 30% of New Zealanders aged 15-18-years classified as overweight or obese [1]. Overweight adolescents are likely to remain overweight or obese throughout the life course, which can have a detrimental influence on their risk of other chronic disease [2]. While there is some evidence that food choice is associated with body composition, the association between food choice and obesity amongst adolescents, in particular, is less clear.

While the relationship between obesity and food choice is not straight forward, this lack of a consistent association may be because most previous work investigating obesity and food choice in adolescents has focused on single foods or food groups [3]. Dietary patterns have been proposed as a solution to investigating the association between food choice and body composition as these analyses allow for the entire diet to be examined in combination, rather than focusing on intakes of single nutrients or food groups. The few studies investigating principal components analysis derived dietary patterns and body composition in children and adolescents have found that those patterns high in fruits and/or vegetables are negatively associated with overweight and obesity [4-6]. When separate analyses have been undertaken for girls and boys, those girls with higher scores for fruit and/or vegetables patterns are less likely to be overweight, whereas the opposite has been found for boys [5]. Patterns high in treat/snack foods have also shown a negative association with obesity [7,8], waist circumference, and waist-to-height ratio [9]. This is not the intuitively expected direction of the association, as these treat/snack food dietary patterns are generally representative of a high energy diet [10].

One factor that may influence the association between obesity and food choice is whether an individual is dieting or trying to lose weight. Food consumption patterns differ between dieters and non-dieters and previous work in adolescent populations found that, compared to non-dieters, dieters report lower consumption of snack foods and sugar-sweetened beverages [11,12] and higher consumption of fruits and vegetables [11]. In addition, research into food choice has found dieting status to be a stronger predictor of food choice than weight status in adolescents [13]. For these reasons, current dieting status may mask the true association between food choice and body composition as those dieting may have a high BMI but also be more knowledgeable about their food choice and are either reporting low frequencies of the foods they think they should avoid, or are actually consuming less of these foods [14].

Furthermore the inconsistencies in the associations between food choice and body composition may be related to the actual body composition measures used in previous studies. While BMI is one of the most commonly used body weight indices, as weight and height are relatively easy and inexpensive to measure, it is not without limitations. BMI does not distinguish between fat mass and lean mass, and the increased risk associated with CVD is thought to be related to excessive body fat rather than weight itself [2]. Therefore not isolating fat mass from overall body mass may mask, or attenuate, an association with dietary intake. A solution is to measure fat mass using measures such as bio-electrical impedance analysis (BIA) or duel-energy x-ray absorptiometry (DXA).

Therefore, the aims of the present study were to investigate (i) the association between dietary patterns and several measures of body composition in adolescents from Otago, New Zealand; (ii) whether there were differences in these associations between girls and boys; and (iii) whether the associations between dietary patterns and body composition were moderated by current dieting status.

## Methods

### Study design and participants

The present study analysed data collected as part of the Otago School Students Lifestyle Survey Two (OSSLS2), a cross-sectional study conducted from February to June 2011 in the Otago province of New Zealand. Data were collected on food choice, psychosocial correlates of diet, eating behaviours, and body composition. Secondary schools in the Otago area were invited to take part in the study. Randomly selected classes from school years 11 to 13 (age 14 to 18 years) from each school were invited to take part, with one class from each school year in smaller schools and up to four classes in larger schools invited. In the week before the projected data collection invited students were given information sheets and consent forms for themselves and their parents. Students were required to sign a consent form in order to participate, while parents were only required to sign a form if they wished their child to opt-out of the study. This study was approved by the University of Otago Human Ethics Committee.

### Survey

The survey was administered online during class time in the schools’ computer rooms. Students supplied information on their date of birth, age, sex, ethnicity and residential address. Ethnicity was categorised into three groups, in accordance with other national New Zealand surveys [1]: ‘Māori’; ‘Pacific Island’; or ‘New Zealand European or Other’ (NZEO). The NZEO group comprised those who identified as New Zealand European, plus other groups where numbers are too small to allow separate analyses e.g. those who identified as Indian, Asian, European. Home residential address was used to assign a neighbourhood area-based New Zealand Deprivation Index (NZDep06) score for each participant. NZDep06 combines nine variables from the 2006 New Zealand census which reflect eight dimensions of deprivation, including income, owning a house, access to a car [15]. NZDep06 ranges from 1 (least deprived) to 10 (most deprived). This variable was collapsed into 5 categories. NZDep06 was only used to describe characteristics of the participants, as this information was not available for all participants. School decile is determined by the deprivation level, as measured by NZDep06, of students attending the school, with the lowest decile rating reflecting the 10% of schools nationwide with students mostly from high deprivation areas, and the highest decile rating reflecting the upper 10% of schools with students from mostly low deprivation areas. School decile was divided into ‘Middle’ (Deciles 5 to 8) and ‘High’ (Deciles 9 and 10).

Food consumption was assessed, as part of the online survey, using a food frequency questionnaire (FFQ) adapted from the Health Behaviour in School Children study (HBSC) [16]. This questionnaire was pretested in a sample of adolescents from Otago before use in OSSLS2 and showed good repeatability and relative validity [17]. Participants were asked ‘*On average, how many times a week do you usually eat or drink any of the following foods?*’. The food items assessed were ‘fruit’, ‘vegetables’, ‘sweets’ (e.g. jelly beans, marshmallows, etc.), ‘chocolate confectionery’ (e.g. chocolate, Moro, Crunchie, etc.), ‘standard milk’ (≈ 3.3% total fat), ‘other milk’ (low fat, rice, soy), ‘cheese’, ‘breakfast cereals’, ‘white bread’, ‘brown/wholegrain bread’, ‘potato crisps’, ‘hot chips/fries’, ‘artificially-sweetened ‘soft-drink’ (e.g. Diet Coke, Coke Zero, etc.), ‘regular soft-drink’ (e.g. Coke, Pepsi, etc.), ‘energy drinks’ (e.g. Red Bull), ‘sports drink’ (e.g. Powerade), ‘fruit juice/fruit drink’ and ‘alcohol’. Regular soft-drink, energy drinks and sports drinks were collapsed into one food group for this analysis called sugar-sweetened soft-drinks.

Dieting status was examined using the HBSC question ‘*At present, are you on a diet or doing something else to lose weight?*’. The response options were ‘No, I am happy with my weight’; ‘No, but I should lose some weight’; ‘No, because I need to put on weight’ and ‘Yes’. This question was dichotomized into 0 (non-dieters) for those participants who answered ‘No, I am happy with my weight’ or 1 for all other responses, “dieters; or those who think they should make changes to their weight”.

### Body composition

Height was measured twice to the nearest millimetre, with a calibrated portable stadiometer (University of Otago, NZ). Fat mass (in kilograms) and fat-free/lean mass (in kilograms) were estimated with BIA using a calibrated segmental machine (BC-418, Tanita Corporation, Japan), which also measured weight (in kilograms). Mid-point waist circumference, between the lower costal border of the last rib and the top of the iliac crest, was measured twice to the nearest millimetre with a body composition non-elastic tape (Seca, Germany). A third measure was taken if the initial two readings differed by more than 0.5cm. All body composition measures were taken by trained research assistants.

BMI was calculated as weight (in kilograms) divided by height squared (in metres). Age-specific BMI z-scores, using the 2007 World Health Organisation method [18], were determined, as were thinness, normal-weight, overweight and obese categories using the 2012 International Obesity Task Force cut-points [19]. Because of the low prevalence of obese individuals in the current sample, the overweight and obese groups were combined, hereafter referred to as overweight. Likewise, those with a low BMI-for-age (thinness) were combined with the normal-weight category. Fat mass index (FMI) was determined as fat mass (in kilograms) divided by height squared (in metres), fat-free mass index (FFMI) was calculated as lean mass (in kilograms) divided by height squared (in metres). The waist-to-height ratio was calculated as waist circumference (in centimetres) divided by height (in centimetres).

### Statistical analysis

Participants were excluded if they had incomplete demographic information, as this was the first section of the survey. Participants were also excluded if their responses indicated that the survey was not completed properly. Reasons for exclusion included clicking patterns (e.g. selection of extreme left or right answer options for all questions), contradictory responses to similar questions, or giving several unrealistic answer options. Only participants who had complete data for all body composition variables were included in the final analysis. Missing data were imputed only for questions consisting of four or more sub-questions and only for those who had completed at least 75% of the sub-questions. For example the FFQ was considered a question, with each food item considered a sub-question. Missing answers to sub-questions were imputed with the worst-case scenario response. For the current analyses, the only data imputed were for the FFQ question. Of the 681 participants who completed at least 75% of the FFQ, data was imputed for a maximum of two participants for any given sub-question. Dietary pattern scores were derived using Principal Components Analysis (PCA). The PCA was run with all 16-food groups, entered as raw frequencies using varimax orthogonal rotation. Factors were retained on the basis of eigenvalues >1.0, a scree plot of the eigenvalues and interpretability of components [20]. Variables with factor loadings ≥ 0.3 and ≤ −0.3 were considered significant when naming patterns. The patterns produced were transformed to remove skew and converted to z-scores, so factor scores had a standardised mean of zero. All participants received a score for all three patterns, with positive scores indicating high consumption of foods associated with that pattern and negative scores indicating lower consumption, for example the higher the ‘Fruits and Vegetables’ score, the higher an individual’s frequency of consumption for fruit, vegetables, cheese, and brown/multigrain bread.

Body composition values were examined as the dependent variables, while the dietary pattern scores were used as the independent variables. Gaussian family generalized estimating equations, with robust standard errors, were used to account for the complex sample in order to ensure results are representative of the population in the sampled area, with schools as the clustering units. For every unit increase in the dietary pattern score, the beta coefficient reflects the expected change in the body composition outcome. As some of the models were not linear in their associations, some outcome variables (waist circumference, waist-to-height ratio, FMI, and FFMI) were logarithmically transformed. For those models with transformed outcomes, the exponentiated beta coefficient reflects the percent change in the geometric mean of the body composition outcome for every unit increase in the dietary pattern score.

Several models were used to examine associations of interest. Model one was adjusted for age only. As sex [21], SES [22] and ethnicity [23] have been found to be associated with food choice, model two included these factors. Model two was repeated using different individual measures of deprivation, including NZDep06, household crowding and car ownership. As these different markers of SES made no difference to effect sizes, and school decile was the only deprivation variable available for every participant, school decile was used in the final analysis. Model three consisted of model two plus a dieting status by dietary pattern interaction variable, while model four consisted of model two plus the relevant sex by dietary pattern interaction. As differences between dietary patterns and overweight have been found between boys and girls [5,7], model five was run separately for boys and girls and adjusted for age, school decile, and ethnicity. Model results in tables are presented with no interaction terms. When a significant interaction was found this was indicated with a superscript in the table while the relevant models including the interaction terms were presented visually with figures. A two-sided *P*-value of <0.05 was considered statistically significant. Statistical analyses were undertaken using Stata statistical software package version 12.0IC (StataCorp, College Station, TX, USA).

## Results

### Descriptive analysis

Eighteen Otago secondary schools were invited to take part in the OSSLS2 study, of which five schools declined to participate and two schools could not accommodate the time frame, providing access to 933 eligible participants, of which 155 students were absent on collection day and 48 refused to participate. Of the 730 participants who completed the study, 17 participants were excluded as responses indicated the survey was not completed properly and 32 participants were excluded from analysis due to incomplete data. OSSLS2 participants represented 25% of all eligible students in the sampling region. The school deciles for the 11 participating schools ranged from five to ten. This is representative of the range for secondary schools within the Otago region, as of July 2010 (the latest available data), with the exception of one small area school which had a decile rating of four. Complete demographic, food, and anthropometric data were available for 93% of those who participated in the survey.

The majority of participants were of ‘New Zealand European or Other’ ethnicity (90%), most of whom identified themselves as ‘New Zealand European’ (75% of total sample). This category also included those self-identified as Asian (7% of total sample), other European for example English (7% of total sample), and African (0.7% of total sample). Participants were aged from 14 to 18 years, with a mean age of 15.8 (SD 0.9) years. The highest proportion of students were from the least deprived neighbourhoods (Table 1). Most participants were of ‘normal-weight’ (Table 1), of whom 38 had a low BMI-for-age. Of the 181 classified as ‘overweight’ (Table 1), 42 were obese (data not shown). Only ethnicity and dieting status were found to differ significantly by BMI status (*P* = 0.001 and *P* < 0.001, respectively). As expected, body composition differences were seen between the sexes, and overweight individuals had less healthy values for other body composition measurements (data not shown).

**Table 1:** Demographic characteristics of participants in the OSSLS2 by BMI classification

	**BMI classification**					
	**Total sample**		**Normal**		**Overweight**	
	**n**	**(%) **^**1**^	**n**	**(%) **^**1**^	**n**	**(%) **^**1**^
All	681		500	(73)	181	(27)
Sex						
*Girls*	297	(44)	217	(73)	80	(27)
*Boys*	384	(56)	283	(74)	101	(26)
Ethnicity						
*NZEO*	612	(90)	460	(75)	152	(25)
*Māori*	59	(9)	37	(63)	22	(37)
*Pacific*	10	(1)	3	(30)	7	(70)
School Year						
*11*	300	(44)	217	(72)	83	(28)
*12*	206	(30)	155	(75)	51	(25)
*13*	175	(26)	128	(73)	47	(27)
School Decile						
*Middle*	268	(39)	187	(70)	81	(30)
*High (Least deprived)*	413	(61)	313	(76)	100	(24)
NZDep06						
*1 (Least deprived)*	278	(42)	204	(73)	74	(27)
*2*	130	(20)	104	(80)	26	(20)
*3*	114	(17)	79	(69)	35	(31)
*4*	99	(15)	72	(73)	27	(27)
*5 (Most deprived)*	43	(6)	30	(70)	13	(30)
Dieting Status						
*Not dieting, happy with weight*	228	(40)	201	(88)	27	(12)
*Not dieting, need to gain weight*	83	(14)	73	(88)	10	(12)
*Not dieting, should lose weight*	191	(33)	107	(56)	84	(44)
*Currently dieting*	77	(13)	48	(62)	29	(38)

OSSLS2 Otago School Students Lifestyle Survey Two; NZEO New Zealand European and Other; NZDep06 New Zealand Deprivation Index.

^1^ Row percentage, unadjusted for confounders.

### Dietary pattern analysis

PCA produced three components, which explained 46.7% of the total variance within the food data (Table 2). The first component produced was a ‘Treat Foods’ pattern, loading highly for sweets, chocolate confectionery, potato crisps, hot chips/fries, artificially-sweetened soft-drinks, and sugar-sweetened soft-drinks. The second component produced was a ‘Fruits and Vegetables’ pattern, which loaded highly for fruit, vegetables, cheese, and brown/multigrain bread. The third component loaded highly for standard milk, other milks, breakfast cereal, and white bread, and was labeled the ‘Basic Foods’ pattern.

**Table 2:** Factor loadings of various food items in the three principal dietary component patterns identified (loadings above 0.3 are shown in bold) in Otago adolescents using a food frequency questionnaire adapted from the Health Behaviour in School-aged Children Survey

**Food item**	**Treat foods**	**Fruits & Vegetables**	**Basic foods**
(variance explained)	(21.15%)	(15.15%)	(10.37%)
Fruit	−0.06	**0.49**	0.01
Vegetables	−0.08	**0.47**	0.09
Sweets	**0.40**	0.08	−0.00
Chocolate Confectionary	**0.41**	0.13	−0.10
Standard Milk	−0.00	0.05	**0.63**
Other milks	0.05	0.30	**−0.48**
Cheese	0.16	**0.33**	−0.03
Breakfast Cereal	−0.02	0.29	**0.35**
White Bread	0.11	0.06	**0.39**
Brown/Multigrain Bread	0.08	**0.41**	−0.15
Potato Crisps	**0.31**	0.03	0.19
Hot Chips/Fries	**0.39**	−0.10	0.07
Artificially-sweetened Soft-Drink	**0.34**	−0.11	−0.12
Sugar-sweetened Soft-Drink	**0.42**	−0.13	0.01
Fruit Juice	0.20	0.09	0.08
Alcohol	0.18	−0.09	−0.05

### Multivariate analysis

#### Total sample analysis

A significant association was found between the ‘Basic Foods’ pattern and FMI only. For every SD increase in the ‘Basic Foods’ score we expect to see a decrease of 3.58% (95% CI −6.14, -0.94) in the geometric mean for FMI (Table 3).

**Table 3:** Associations between dietary patterns and body composition measures of participants of the OSSLS2

	**Total sample (n = 681)**				**Girls only (n = 297)**		**Boys only (n = 384)**	
	**Age adjusted model **^**1**^		**Multivariate adjusted model **^**2**^		**Multivariate adjusted model **^**3**^		**Multivariate adjusted model **^**3**^	
	**β**	**(95% CI)**	**β**	**(95% CI)**	**β**	**(95% CI)**	**β**	**(95% CI)**
BMI z-score ^4^								
*Treat Foods*	−0.01	(−0.09, 0.07)	−0.02	(−0.10, 0.06) ^7^	−0.05	(−0.19, 0.08)	0.02	(−0.16, 0.20)
*Fruits & Vegetables*	−0.09	(−0.21, 0.02)	−0.10	(−0.22, 0.01)	−0.01	(−0.12, 0.10)	−0.15	(−0.33, 0.03)
*Basic Foods*	0.02	(−0.06, 0.09)	0.00	(−0.07, 0.07)	0.05	(−0.07, 0.17)	−0.02	(−0.09, 0.05)
Waist Circumference ^5^								
*Treat Foods*	−0.46	(−1.61, 0.70)	−0.75	(−1.93, 0.45) ^7^	−0.61	(−2.39, 1.19)	−0.83	(−2.01, 0.37)
*Fruits & Vegetables*	−0.25	(−1.00, 0.51)	−0.16	(−0.92, 0.60)	−0.08	(−1.40, 1.25)	−0.06	(−0.77, 0.66)
*Basic Foods*	−0.28	(−1.29, 0.74)	−0.62	(−1.63, 0.39)	0.33	(−1.46, 2.15)	−1.17	(−1.96, -0.39) ^6^
Waist : Height ^5^								
*Treat Foods*	−0.83	(−2.01, 0.37)	−0.75	(−1.96, 0.47) ^7^	−0.44	(−2.28, 1.44)	−0.96	(−1.96, 0.04)
*Fruits & Vegetables*	−0.46	(−1.19, 0.29)	−0.59	(−1.39, 0.22)	−0.42	(−1.69, 0.87)	−0.63	(−1.56, 0.30)
*Basic Foods*	−0.65	(−1.57, 0.28)	−0.58	(−1.51, 0.35)	0.47	(−1.27, 2.25)	−1.20	(−1.81, -0.58) ^6^
Fat Mass Index ^5^								
*Treat Foods*	−6.10	(−10.65, -1.33) ^6^	−3.57	(−7.69, 0.74)	−2.72	(−7.45, 2.25)	−4.12	(−9.38, 1.44)
*Fruits & Vegetables*	−0.23	(−2.21, 1.78)	−2.55	(−5.12, 0.09)	−0.55	(−4.13, 3.15)	−3.71	(−6.53, -0.81) ^6^
*Basic Foods*	−5.94	(−8.83, -2.97) ^6^	−3.58	(−6.14, -0.94) ^6^	−1.35	(−5.20, 2.66)	−4.90	(−7.33, -2.41) ^6^
Fat-Free Mass Index ^5^								
*Treat Foods*	−0.08	(−0.97, 0.82)	−0.81	(−1.67, 0.06)	−0.94	(−2.52, 0.66)	−0.49	(−1.69, 0.73)
*Fruits & Vegetables*	−0.04	(−0.65, 0.58)	0.33	(0.10, 0.77)	0.66	(−0.25, 1.58)	0.29	(−0.19, 0.78)
*Basic Foods*	0.36	(−0.61, 1.34)	−0.49	(−1.21, 0.24) ^8^	0.53	(−0.82, 1.89)	−1.15	(−1.84, -0.45) ^6^

OSSLS2 Otago School Students Lifestyle Survey Two; Waist : Height = Waist Circumference (cm)-to-Height (cm) ratio.

^1^ GEE regression controlled for age (Model 1).

^2^ GEE regression controlled for age, sex, school decile, and ethnicity (Model 2).

^3^ GEE regression controlled for age, school decile, and ethnicity (Model 5).

^4^ Expected change in mean BMI z-score for every unit change in dietary pattern score.

^5^ Expected percentage change in geometric mean of body composition variable for every unit increase in dietary pattern score.

^6^ Significantly different from zero, *P* < 0.05.

^7^ Significant dieting status by dietary pattern interaction observed in Model 3 (See Figure 2 for model results that include a significant interaction term).

**Figure 1:**
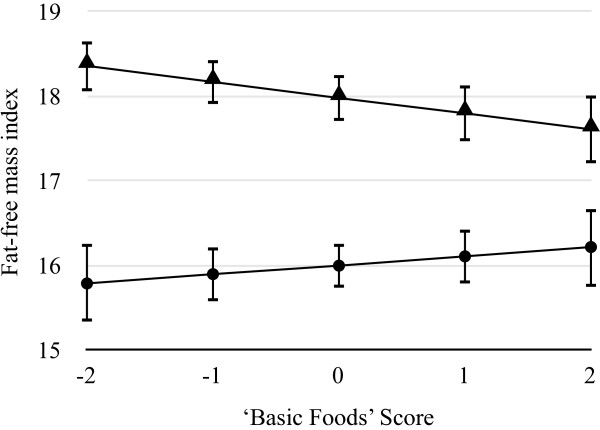
**Visual representation of the significant interaction between sex and the ‘Basic Foods’ dietary pattern for the fat-free mass index.** Figure displays result (with 95% confidence intervals) for a 16-year old student from a high decile school. Interaction statistically significant when adjusted for age, school decile, and ethnicity (*P* < 0.05). ● Female ▲ Male

^8^ Significant sex by dietary pattern interaction observed in Model 4 (See Figure 1 for model results that include a significant interaction term).

#### Separate sex analysis

With increasing ‘Basic Food’ scores there was a significant decrease in waist circumference, waist-to-height ratio, FMI, and FFMI for boys (Table 3). For every SD increase in the ‘Basic Foods’ pattern, there was a decrease of 1.17% (0.92 cm) (95% CI −1.96, -0.39) in the geometric mean for waist circumference for boys. No association between the PCA components and any of the body composition measures was found to be significant for girls (Table 3). A significant pattern by sex interaction was found for the ‘Basic Food’ pattern and FFMI (Figure 1). The results presented are for a 16-year-old student at a high decile school, as the direction and magnitude of the slopes did not differ between the high and middle decile schools. An SD increase in the ‘Basic Foods’ score for an average sized (69.4kg, 1.76m) boy would be associated with a decrease of 2.7kg in lean mass.

#### Dieting status analysis

Information on dieting status was only available for 579 participants. Of these 40% were happy with their weight (Table 1). More girls (24%), than boys (6%), reported currently being on a diet or doing something to lose weight (*P* < 0.001), while more boys (23%), than girls (2%), reported they needed to gain weight (*P* < 0.001) (data not shown). The 102 participants with no information on current dieting status did not differ significantly from those with dieting information with regard to body composition data or mean dietary pattern scores (data not shown). When analyses were repeated for all models with only those who had dieting data, effect sizes were unchanged (data not shown).

**Figure 2:**
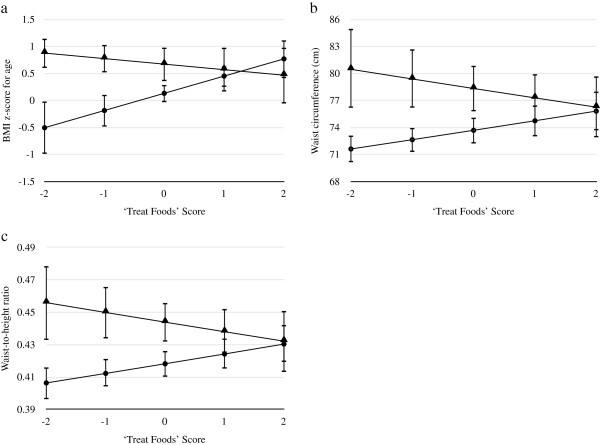
**Visual representation of the significant association between a) BMI z-score; b) waist circumference; and c) waist-to-height ratio and the ‘Treat Foods’ dietary pattern stratified by dieting status.** Figure displays results (with 95% confidence intervals) for a 16-year old (mean age) boy from a high decile school. Interaction statistically significant when adjusted for age, sex, school decile, and ethnicity (*P* < 0.05) ● ‘Not dieting, happy with weight’ ▲ ‘dieters; or those who think they should make changes to their weight’

Further analysis was undertaken to investigate whether dieting moderated the association between the PCA patterns and body composition measures. A significant current dieting status by ‘Treat Foods’ interaction was found for BMI z-score, waist circumference, and waist-to-height ratio (Figure 2). As the direction and magnitude of the slopes did not differ between sexes or school decile, the results are presented for a 16-year-old boy at a high decile school. The results indicate that with increasing ‘Treat Foods’ score BMI z-score, waist circumference, and waist-to-height ratio increase for non-dieters, but decrease for “dieters; or those who think they should make changes to their weight” (Figure 2).

## Discussion

The current study adds to the limited literature investigating PCA derived dietary patterns in adolescents. The results indicate that adolescents who had higher scores for the ‘Fruits and Vegetables’ pattern and ‘Basic Foods’ pattern had healthier body compositions. While significant results were seen for measures of central adiposity (waist circumference and waist-to-height ratio), the results were most pronounced for FMI, a measure of total adiposity.

### Sex differences

Our results are consistent with previous studies in finding different effect sizes between the sexes for the association between dietary patterns and body composition [5,7]. While the current study found greater effect sizes for boys compared to girls, previous results suggest girls have lower odds of being overweight with higher adherence to vegetable patterns [5], and boys show lower odds of overweight and obesity with increasing treat/snack food adherence [5,7]. These results are congruent with previous work suggesting boys and girls have different food preferences [24].

### Dieting status

This is the first study to have investigated relationships between dietary patterns, dieting status and body composition. Our initial analyses indicated a non-significant negative association between the ‘Treat Foods’ score and body composition measures, a finding similar to those seen in previous studies in adolescent populations [5,7-9]. This association is unexpected as dietary patterns high in treat/snack foods have been found to be high in energy [10], so consuming more of these foods could result in less healthy body compositions. Once dieting status was added to the models, a positive association between the ‘Treat Foods’ dietary pattern and body composition was seen. It is likely in previous studies the true association may have been masked because dieting status was not examined.

Our findings are in agreement with similar work by Utter *et al.*[25], who have previously examined food choice in New Zealand adolescents. Although this study population was mainly overweight and obese and of Pacific Island descent, the direction of their associations were congruent with our results. They found that adolescents not wanting to change their weight had higher BMI values, while those who reported currently trying to lose weight had lower BMI values with increasing frequency of consumption of high sugar snack foods and fast foods [25]. The negative association seen between treat/snack foods and body composition in the dieters, in our study and by Utter *et al.*[25], could possibly be a result of dieters reducing their consumption of treat/snack foods as a means of dietary restraint, or as a consequence of social desirability. Whilst we can only speculate that this is the case in this cohort, future research could investigate if this is the case.

### Body composition differences

In the current study, the associations between the ‘Fruit and Vegetable’ and ‘Basic Food’ patterns and body composition were most pronounced for measures of total adiposity (FMI), but not for BMI or central adiposity as indicated by waist circumference and waist-to-height ratio. These results potentially indicate change in fat mass may be more important than changes in overall body composition when examining food choice. Previous research suggests this may be because the environment has been found to influence fat mass more than lean mass [26], and our results suggest this is the case for the food choice environment. While the ‘Treat Foods’ dietary pattern was not associated with total adiposity (FMI) it was found to be associated with BMI and central adiposity, when stratified by dieting status. It may be that while dieters are unaware of their specific body composition, they are dissatisfied with their body size [27] and as a means of reducing their weight dieters have been found to reduce intakes of treat/snack foods compared to non-dieters [12,27].

The current study found no association between ‘Fruits and Vegetables’ or ‘Basic Foods’ patterns and BMI. While BMI is a measure of excess weight and not necessarily excess body fat, it has been shown to have good specificity [28], and may be a good proxy for fat mass in obese adolescents [18]. However, in normal-weight or overweight adolescents higher BMI may be more reflective of increased lean mass rather than fat mass [18]. For this reason BMI may not have been a good proxy for body fat in the current study, as our sample has a low prevalence of obese individuals, and this may explain why the FMI and FFMI were significantly associated with the dietary pattern outcomes and BMI was not. However, associations were seen between ‘Treat Foods’ score and BMI when dieting status was accounted for. This finding may be explained by BMI being a better proxy of fatness in obese individuals [18], and obese individuals in the current study were more likely to diet or be unhappy with their weight.

### Strengths and limitations

All body composition measurements are subject to error. BIA estimates fat and lean mass from population based equations. However, previous research in New Zealand found that adolescent BIA measurements obtained using the same equipment as in this study are highly correlated with DXA measurements [29]. An ISAK (The International Society for the Advancement of Kinanthropometry) qualified instructor trained all research assistants in the body composition measurements and standard operating procedures were followed in order to reduce this error.

The current sample of adolescents is not nationally representative due to a higher proportion of participants of ‘New Zealand European or Other’ ethnicity, from higher socio-economic groups, and fewer overweight or obese individuals than the New Zealand population [1]. However, as the majority of participants are of ‘New Zealand European or Other’ ethnicity, the results of the current study are likely to be applicable to the majority of New Zealand adolescents.

A commonly cited problem with dietary patterns is their lack of generalisability, as the patterns produced tend to be population-specific. While this is the first study to describe dietary patterns in 15-18-year olds in New Zealand, the general nature of our patterns are comparable with other study populations. A Finnish study in adolescents, which used a similar version of the HBSC FFQ (a non-quantitative FFQ), found patterns very similar to our own [9]. The use of dietary patterns has allowed for a more comprehensive approach to assess dietary intake than single nutrients.

A further limitation may be that the FFQ used in this study only assessed frequency of intake, not amount, so the role of particular foods cannot be assessed independently of energy intake. As a consequence we can only conclude that any association with body composition is due to the reported frequency of consumption and not as a result of increased energy intake. The lack of a significant association between dietary patterns and body composition for girls could be a true non-association, or girls may have greater food awareness and as a result are more controlled with regards to food than boys [30]. Nonetheless, the FFQ used in this study has been shown to be valid for ranking participants with reasonable validity [17], and dietary pattern analysis used in the current study allowed for a comprehensive analysis of the effects of the diet. Another limitation of the current study is that we were unable to adjust for physical activity in these analyses, therefore we cannot rule out the possibility that those on a diet were also more active.

This study has many strengths, particularly the inclusion of several body composition measures, including those specific to fat mass. All questionnaires used were pilot tested and validated in the sample population. The current study had a high participant response rate with 78% of those invited agreeing to take part in the study, which is higher than comparable studies with similar findings, where response rates ranged from 53–66% [7-9,25]. The investigation of current dieting status as a moderating variable was a novel approach which showed that the association between treat/snack food type patterns and body composition differed by dieting status.

## Conclusions

In conclusion, significant associations were found between dietary patterns and the indices related specifically to body fatness and lean mass. As we found that dieting status elucidates the association between ‘Treat Foods’ and body composition, future studies should include information on dieting status when investigating the association between food choice and body composition. Future research could include longitudinal studies in adolescents in order to determine the stability of these patterns into adulthood and their continued relationship with body composition. Despite the small effect sizes in body composition seen in the current study, they may still have important public health implications. Our results support current national New Zealand guidelines to eat more fruits and vegetables and wholegrain bread and to try limiting intake of high fat high sugar treat/snack foods in order to achieve and maintain a healthy body composition in adolescence. These messages may be particularly important for boys.

## Abbreviations

CVD: Cardiovascular disease; BMI: Body mass index; FMI: Fat mass index; PCA: Principal components analysis; OSSLS2: Otago School Students Lifestyle Survey Two; SES: Socio-economic status; NZDep06: New Zealand Deprivation Index; FFQ: Food frequency questionnaire; HBSC: Health Behaviour in School-aged Children; BIA: Bioelectrical impedance analysis; FFMI: Fat-free mass index; DXA: Duel-energy X-ray Absorptiometry.

## Competing interests

The authors declare that they have no competing interests.

## Authors’ contributions

ASH, PMLS, and KEB were responsible for conception of this particular study and performing data analyses. ASH drafted the initial manuscript. PMLS and KEB are the principal investigators for the overall OSSLS2 project and were responsible for conception and design of the project and oversaw questionnaire design, data collection and processing. ASH and JEW contributed to the design of the project, including questionnaire design, and data collection and processing. WRP also contributed to study design. All authors provided critical review and revision of the manuscript and have read and approved the final version of the manuscript.
